# How Do Pharmaceutical Companies Overcome a Corporate Productivity Crisis? Business Diversification into Medical Devices for Growth Potential

**DOI:** 10.3390/ijerph18031045

**Published:** 2021-01-25

**Authors:** Yoonje Euh, Daeho Lee

**Affiliations:** Department of Interaction Science, Sungkyunkwan University, Seoul 03063, Korea; yoonje.euh@bd.com

**Keywords:** pharmaceutical company, business diversification, medical device, technical efficiency, meta-frontier analysis

## Abstract

The purpose of this study was to analyze the performance of pharmaceutical companies’ business diversification into medical devices in terms of their technical efficiency (TE) as compared to that of traditional pharmaceutical companies. For a total of 174 externally audited pharmaceutical companies engaged in the drug product business between 2008 and 2019, pharmaceutical companies were classified into two groups according to medical device business diversification. The TE of pharmaceutical companies that diversify the medical device business was lower than that of traditional pharmaceutical companies. However, in terms of the meta-technology ratio (MTR) calculated using meta-frontier analysis, pharmaceutical companies diversified into medical devices showed higher MTR than the traditional pharmaceutical company group. The results imply that the corporate performance growth potential of traditional pharmaceutical companies is lower than that of pharmaceutical companies that have diversified into the medical device business.

## 1. Introduction

Health issues caused by cancer, diabetes, population aging with low birth rate, infectious diseases, etc. are topics of interest to many people. The importance of the medical device and pharmaceutical industries has been emphasized due to infectious diseases such as influenza, Middle East respiratory syndrome, and coronavirus viral disease 2019 (COVID-19). In the case of COVID-19, the virus has serious adverse effects not only on human health but also the social economy. Thus, a diagnostic test device and a vaccine for the disease are being devised and demanded in countries around the world [1,2,3]. This is because medical devices and medicine for patient treatment are directly connected to each other. However, despite the importance of these industries, pharmaceutical companies are under significant pressure between the consumer associations that demand inexpensive products with good efficacy and investors who pursue high performance and profit [4]

Interestingly, research and projects related to the pharmaceutical industry over the past two decades have been invested in discovering new drugs to generate profit [5]. However, as the cost for basic research and development (R&D) is gradually increased, much research and many projects at pharmaceutical companies were stopped [6]. Furthermore, poor R&D led to a decrease in the pharmaceutical industry’s productivity. Specifically, changes in the industrial structure such as the rise of the biotechnology field contributed to the increase in R&D costs of pharmaceutical companies [7]. As productivity declines continue, the pharmaceutical industry is facing unprecedented industrial challenges and surveillance with the extinction of monopoly products and the reduction of pipelines caused by patent expiration [8].

To overcome the crisis of productivity decline, pharmaceutical companies first made an effort to improve the process for finding new drug candidates [9]. When productivity was significantly lowered, R&D through outsourcing was boldly chosen as an alternative to reduce costs [10]. Second, pharmaceutical companies aimed at increasing overall corporate productivity by improving the pharmaceutical manufacturing process [11] and optimizing the supply chain with inventory management [12]. These efforts in internal processes did not lead to improved R&D process for a new drug or higher profit that could raise corporate performance, because the causes of productivity decline in pharmaceutical companies are diverse and complex [13].

Another effort of pharmaceutical companies to overcome the crisis was business diversification [14]. For example, the number of mergers and acquisitions for the pharmaceutical and biotechnology industry in 2018 recorded a total of 1438 and a total volume of $339.6 billion as the highest in the last decade. The medical device industry, which is indicated as a major factor in the decline of the pharmaceutical industry [15], has become a major target of business diversification from pharmaceutical companies. Interestingly, at this time, the number of acquisitions in the healthcare industry (131 cases), including medical devices, was the second highest after acquisitions of the homogeneous sector (449 cases) followed by distribution/logistics (57 cases) and the information and communication sector (30 cases) [16]. Pharmaceutical companies are highly interested in diversifying the medical device business because both medical device and drug are used by patients or doctors as end users for clinical purpose, and the distribution environment of products looks the same. The phenomenon by which multinational medical device companies are gradually developing and launching medical devices that contain medicines can also be attributed to the fact that the business environment is similar [17]. For these reasons, many pharmaceutical companies are expecting to raise overall corporate performance by improving their productivity through the business diversification of medical devices because of the productivity crisis.

However, there are many discussions on how merge and acquisition (M&A) or business diversification will affect corporate performance due to complex factors such as market characteristics of industry and understanding of product, conflicting regulations, organization, etc. as well as additional cost in the process [18,19,20]. In terms of the pharmaceutical industry, the studies for pharmaceutical companies mainly focused on R&D synergy of M&A with the biotechnology industry [21,22,23]. The integration of biotechnology with a similar R&D environment also presents various uncertainties regarding the improvement of pharmaceutical companies’ performance [23]. When considering these studies, there is still an absence of research on whether the business diversification of pharmaceutical companies into medical devices positively affects their corporate performance in the crisis of productivity.

The pharmaceutical business differs from medical device in the characteristics of the entire business cycle from product R&D through sales. In the traditional pharmaceutical business, when a product is released through a large R&D investment, it continuously generates high profits with improving the supply of raw materials and promoting sales within the protection of patent rights [24]. When compared to pharmaceuticals, the medical device business has a short product life cycle and risk about easy product duplication, so the market competition is overheated due to the low entry barrier and product profits are not very high. Due to these differences, when a pharmaceutical company diversifies its medical device business, the total sales of the company might increase, but the overall performance of the company might decrease. Furthermore, considering additional costs and time, such as new production line, labor, preparation for medical device regulations, and expenses from sales and management, manufacturers should be aware that pharmaceutical and medical device businesses differ not only in their product development and life cycles, but also in the nature of the business and legal factors [25].

This study attempted to determine whether diversifying the medical device business can increase the performance of pharmaceutical companies by analyzing financial data from South Korea. According to the Ministry of Food and Drug Safety (MFDS), the Korean pharmaceutical market in 2019 increased by 5.2% from 2018 (21.24 billion USD) to 22.33 billion USD, ranking 12th in the world (1.6%). Although the Korean pharmaceutical market is growing at a high level as a mature market, overall pharmaceutical companies are facing a decline in corporate performance and a productivity crisis due to the cost of new drug development, patent expiration, regulation, and competition. The phenomenon is common among pharmaceutical companies around the world. For this reason, many pharmaceutical companies in South Korea are expecting to improve corporate performance through diversification of medical device business. South Korea became a country where the trend and phenomenon of pharmaceutical companies that are representatively diversifying medical device business is gradually expanded to overcome the decline in corporate performance and a productivity crisis. For this purpose, this study measured the performance of companies using technical efficiency (TE) indicators. Where sales or productivity is affected by the company size, technical efficiency has the advantage of not being affected by the firm size because it estimates the firm’s production function first and measures technical efficiency according to the distance from the production function. Recently Chung et al. [26], Jo et al. [27], Na et al. [28], and many others measured firm performance using technical efficiency in accordance. Because conventional TE has the disadvantage of not being available for comparison between companies using different production functions, we compared pharmaceutical companies that have expanded their business to medical devices with traditional pharmaceutical companies in terms of a firm’s TE.

## 2. Methodology

### 2.1. Stochastic Frontier Analysis (SFA)

SFA expresses the relationship between input and output factors as a production function and estimates the TE using the frontier production function representing the maximum output from the input. At this time, the TE of a company indicates where the company’s technology level is relatively located, when compared to the efficiency technology level of the frontier production function. The farther the technology level of a company is from the frontier production function represents a lower level of efficiency. The production frontier can be estimated through both nonparametric and parametric methods. In this study, the production frontier for the parametric method was estimated using SFA. Also, FRONTIER *Version 4.1* software provided by Coelli was used for estimation.

According to Battesse and Coelli [29], the model of the stochastic production frontier is assumed as the following Equation (1) to reflect the change of time in efficiency.

(1) $$Y_{it}=f\left(x_{it},\;\beta \right)e^{V_{it}-U_{it}},\;i=1,2,\ldots ,N,\;t=1,\;2,\ldots ,\;T$$

At this time, *Y_it_* is the output of company *i* at time t, *x_it_* is the input vector of company *i* at time *t*, *f* is the production function, β is a vector containing the parameters of the production function, *V_it_* is independent of *U_it_* with a random error with a distribution of $N\left(0,\;\sigma_{v}^{2}\right)$, and *U_it_* is a nonnegative random variable representing the TE of company *i* at time *t*. If *V_it_* is the general random error of the regression equation, then *U_it_* represents the company’s inefficiency. To show that it is always inefficient, *U_it_* itself is not negative and this study assumed that *U_it_* follows a half-normal distribution. Because data from 2008 to 2019 were used, *T* is 12.

From Equation (1), the efficiency, $TE_{it}$, of company *i* at time *t* is given by Equation (2) below.

(2) $$TE_{it}=e^{-U_{it}}=\frac{Y_{it}}{f\left(X_{it},\beta \right)e^{V_{it}}},\;i=1,2,\ldots ,N,\;t=1,2,\ldots ,\;T$$

In general, the Cobb–Douglas function and the translog function are the most widely used SFA production functions. However, in the case of Cobb and Glass, there is a tendency to oversimplify it because the output variable is seen as a linear combination of input variables. Therefore, in this study, we used the translog function. In particular, a random-effects, time-varying production model was used. When assuming a translog type of production function, Equation (1) can be expressed as Equation (3) below.

(3) $$\ln Y_{it}=\beta_{0}+\overset{3}{\underset{m=1}{\sum }}\beta_{m}\ln x_{mit}+\overset{3}{\underset{m=1}{\sum }}\overset{3}{\underset{k\geq m}{\sum }}\beta_{mk}\ln x_{mit}\ln x_{kit}+V_{it}-U_{it}$$

where *x*_1*it*_ represents the amount of capital (K) of the *i*-th company at time *t*, *x*_2*it*_ represents the cost (M) of the *i*-th company at time *t*, and *x*_3*it*_ is the number of workers (L) who receive a salary from the *i*-th company at time *t*. The total assets for K, the number of employees for L, and the cost of revenue for M are used as input variables, and net sales (Y) are used as an output variable in this study.

### 2.2. Meta-Frontier Analysis

Since the TE of a specific company is difficult to compare with other companies using different technologies, comparisons of technological efficiency between each group cannot be performed through traditional SFA. Therefore, to compare the efficiency levels of different groups operating under different technical conditions, we used the meta-frontier production function that wraps the production functions of all groups [30]. From Battese, Rao, and O’Donnell [31], the meta-frontier production function model is defined as follows.

(4) $$Y_{it}^{*}=f\left(x_{it},\;\beta^{*}\right)=e^{x_{it}\beta^{*}},\;i=1,2,\ldots ,N,\;N=\overset{R}{\underset{i=1}{\sum }}N_{j},\;t=1,\;2,\ldots ,\;T,\;s.t.\;x_{it}\beta^{*}\geq x_{it}\beta_{\left(j\right)}\;\mathrm{for}\;\mathrm{all}\;j$$

In this case, $\beta_{\left(j\right)}$ is a vector composed of the parameters of the j-th group’s production function and j indicates each group. In this study, the two groups are traditional pharmaceutical companies that have only produced medicines (*j* = 1) and diversified pharmaceutical companies that also produce medical devices (*j* = 2). $\beta^{*}$ is a vector of unknown variables of the meta-frontier function that satisfies the following equation. From Equation (4) above, the graph of the meta-frontier production function is positioned above the graph of the production frontier function of each group for all periods. The meta-frontier production function becomes an envelope of the frontier functions of each group based on the same technology. For simplicity, if we assume that function *f* in Equation (1) is $e^{X_{it}\beta_{\left(j\right)}}$, Equation (1) can be divided as shown in Equation (5).

(5) $$Y_{it}=e^{-U_{it\left(j\right)}}\times \frac{e^{x_{it}\beta_{\left(j\right)}}}{e^{x_{it}\beta^{*}}}\times e^{x_{it}\beta^{*}+V_{it\left(j\right)}}$$

Dividing both sides of Equation (5) by $e^{x_{it}\beta^{*}+V_{it\left(j\right)}}$ yields the following.

(6) $$\frac{Y_{it}}{e^{x_{it}\beta^{*}+V_{it\left(j\right)}}}=e^{-U_{it\left(j\right)}}\times \frac{e^{x_{it}\beta_{\left(j\right)}}}{e^{x_{it}\beta^{*}}}$$

In Equation (6) above, the right side, $e^{-U_{it\left(j\right)}}$ is the technical efficiency (TE) of Group j and the second is the j group frontier for the meta-frontier function. It is expressed as a ratio of a function, which is called the Technical Gap Ratio or Meta-Technology Ratio (MTR). TE*, representing TE in the meta-frontier function, is calculated by multiplying TE by MTR and can be expressed as Equation (7).

(7) $$TE_{it}^{*}=\frac{Y_{it}}{e^{x_{it}\beta^{*}+V_{it\left(j\right)}}}=TE_{it}\times TGR_{it}$$

There are two methods of measuring the parameters of a meta-frontier function: Linear Programming (LP) and Quadratic Programming (QP). LP is a method of minimizing the sum of the absolute deviation values, and QP is a method of minimizing the sum of the squares of deviations. According to Battese, Rao, and O’Donnell [31], LP and QP are defined as following Equations (8) and (9).

(8) $$\mathrm{LP}:\;\underset{\beta^{*}}{\min}L^{*}=\overset{T}{\underset{t=1}{\sum }}\overset{N}{\underset{i=1}{\sum }}\left|x_{it}\beta^{*}-x_{it}{\hat{\beta }}_{\left(j\right)}\right|,\;x_{it}\beta^{*}\geq x_{it}{\hat{\beta }}_{\left(j\right)}$$

(9) $$\mathrm{QP}:\;\underset{\beta^{*}}{\min}L^{*}=\overset{T}{\underset{t=1}{\sum }}\overset{N}{\underset{i=1}{\sum }}{\left(x_{it}\beta^{*}-x_{it}{\hat{\beta }}_{\left(j\right)}\right)}^{2},\;x_{it}\beta^{*}\geq x_{it}{\hat{\beta }}_{\left(j\right)}$$

Matlab 7.1 software was used to measure the parameters of the meta-frontier function using LP and QP.

## 3. Estimation Results

In this study, actual corporate financial data were secured from the KIS-VALUE database of the National Information and Credit Evaluation. Based on the information from the South Korean Ministry of Food and Drug Safety (MFDS), pharmaceutical companies are divided into traditional pharmaceutical companies and diversified pharmaceutical companies for medical devices according to their import certifications and licenses to manufacture medical device products. Specifically, the number of externally audited Korean pharmaceutical companies that acquire approval to manufacture and import medical devices for general treatment and surgery from the MFDS has increased rapidly since 2008. Because of a novel influenza outbreak in 2009, Korean pharmaceutical companies expanded to new businesses that manufacture, import, and distribute various types of medical devices, from advanced medical devices to diagnostic test kits and instrument. Thus, a total of 174 externally audited pharmaceutical companies in South Korea were identified for the period between 2008 and 2019.

One hundred three traditional pharmaceutical companies that only conducted pharmaceutical business and 71 pharmaceutical companies that diversified into the medical device business were separated into two groups. The total number of samples with fiscal data used in the study was 1028 for traditional pharmaceutical companies and 728 for pharmaceutical companies diversified into medical devices. Table 1 contains details regarding the samples.

FRONTIER 4.1 was used for SFA and meta-frontier analysis (MFA) with MATLAB 7.1 was carried out to verify corporate efficiency. Table 2 shows the estimated frontier production function for each group with meta-frontier production function parameters optimized through LP and QP methods.

Table 3 shows the results of the TE for each group and the value of TE* in the meta-frontier production function with the MTR by using the estimates of the group frontier production function and meta-frontier production function.

As a result, traditional pharmaceutical companies (76.28%) showed higher TE values when compared to diversified pharmaceutical companies for medical devices (59.11%). However, as mentioned earlier, comparisons between groups using different production functions are meaningless. Therefore, the TE of the two groups using different production functions should be compared through MTR. Conversely, a group of diversified pharmaceutical companies for medical devices showed a higher MTR value both with LP and QP (MTR_LP: 94.54%, MTR_QP: 88.82%) when compared to the group of traditional pharmaceutical companies (MTR_LP: 80.39%, MTR_QP: 76.67%).

## 4. Discussion

Traditional pharmaceutical companies have pursued high corporate efficiency through continuous research on pharmaceutical business models in diverse fields such as patents, regulations, distribution, R&D, and manufacturing innovation. With recent advances in marketing strategies and technologies, the pharmaceutical industry has been trying to overcome the decline in productivity by maximizing profits for many years [32,33,34]. Nevertheless, unlike increasing their market size, the problem of decreasing productivity has not yet been solved. For the pharmaceutical industry, this is expected to be a complex cause of the already mature business model, excessive market competition, regulation, and competition with similar businesses such as the biotechnology and medical device industries. Companies might seek to integrate with homogeneous firms to improve performance and expect corporate synergy through the combination and expansion of heterogeneous industries. The pharmaceutical industry is also making an effort to reinforce their business portfolio through mergers and acquisitions and business diversification due to the efficiency decline and low productivity. As mentioned in the introduction, in 2018, a total of 1438 transactions with a total volume of $339.6 billion in global mergers and acquisitions in the pharmaceutical and bio-industry industries were recorded. The number of acquisitions in the healthcare industry including medical devices (131 cases) was the highest after homogeneous industry acquisitions (449) [16].

As mentioned earlier, pharmaceutical companies expect to improve overall productivity by reinforcing R&D pipelines between homogeneous industries [35], while expecting to improve corporate performance through business expansion into different industries. Globally, pharmaceutical companies’ business expansion of the healthcare business including medical devices has gradually increased, and Korean pharmaceutical companies, which are diversifying into the medical device business, also had a total of 31 (17.8%) in 2008. Currently, a total of 71 (40.8%) pharmaceutical companies have gradually increased their number to diversify their medical device business. Thus, the importance of research on how the medical device business affects the performance of pharmaceutical companies has been raised in South Korea.

As a result of this study, the MTR of the pharmaceutical company group that conducted business diversification was higher than that of the traditional pharmaceutical company group. This higher MTR of the diversified group means that the group’s frontier production function is located closer to the meta-frontier production function. The frontier production function is determined by the technology used by the companies in the group and is the set of maximum outputs that the companies can produce. Therefore, the fact that the MTR of the pharmaceutical group that diversified into the medical device business is higher than the MTR of the traditional pharmaceutical group means that the maximum output that can be produced through the same input is higher, that is, it has a higher potential. Interestingly, in the MFA, which is the same production function, both TE* calculated with LP and QP showed higher results than traditional pharmaceutical companies diversifying their medical device business. As described above, TE* can be calculated as the multiplication of TE and MTR. In traditional pharmaceutical companies, although the MTR was lower than that of the pharmaceutical company group that diversified into the medical device business, the TE was much higher than that of the group. Thus, the TE* was higher. This means that traditional pharmaceutical companies are exhibiting higher efficiency under the current production function, but their potential is lower than that of pharmaceutical companies that have diversified into the medical device business.

Based on research results, policy makers and corporate decision makers as well as future study should consider the following implications of this study for sustainable management in the pharmaceutical industry and the medical device industry.

First, in terms of theoretical implications, while most of the existing studies for decades focused mainly on improving the R&D process of the pharmaceutical industry, the industry has been establishing diverse strategies to grow the productivity and performance potential of companies such as the business diversification of medical device. Future studies should be able to fill the blanks in these relevant research topics and provide new perspectives to improve the performance of pharmaceutical companies.

Second, in terms of practical implications, the interaction between doctors and pharmaceutical companies is known to influence the prescription of medicines [36]. Although doctors are users, they have a large portion of the patents for medical devices [37]. Since they have already been involved from the earliest stages of development in clinical trials [38], doctors are interested in not only medicines but also medical devices. Furthermore, since the trend of the treatment process is rapidly changing due to the development of medical devices, the direction of drug development and productivity can be flexibly modified. This is the reason why medicines and medical technology are complementary to each other and technology advancement is simultaneously progressing. Therefore, business diversification into medical devices seems to increase the potential for corporate performance by improving the technological efficiency of pharmaceutical companies.

Third, in terms of policy implications, policy makers considering and reviewing business promotion of the pharmaceutical industry should be encouraged to review diversified business models to improve corporate performance and potential. Furthermore, R&D for new drugs must be continuously conducted by promoting corporate policy with business portfolio expansion of medical device with complicated crisis of productivity.

## 5. Conclusions

The medical device business clearly differs from the pharmaceutical business in terms of the product production cycle from R&D to product launch and follow-up management. However, when we examined the two businesses from the perspective of healthcare providers and users, not from manufacturers or regulatory agencies, medical devices and medicines in treatment are complementarily connected. In other words, both products are developed and used for the purpose of treatment based on the interaction between the doctor and patient. In conclusion, when compared to traditional pharmaceutical companies, pharmaceutical companies diversified into the medical device business are dominating the traditional pharmaceutical companies in the trends of treatment process and the contact point of customers with market changes. Therefore, these advantages appear to improve overall corporate performance growth potential.

As the pharmaceutical industry may experience a decline affected by major developments in the medical device industry and the biotechnology industry, it is necessary to take insights on multiple businesses and have a view of political decision for pharmaceutical industry promotion. Policy makers should not encourage companies to carry out one business and a similar industry as their conservative general policy.

This study has the following limitations. First, since this study used data from externally audited companies for the period between 2008 and 2019, smaller companies with low total assets were excluded from the group. Second, different variables that could affect the TE of pharmaceutical companies were not considered as well as business diversification. Third, the study is insufficient to provide global implications because it analyzed data only from Korean pharmaceutical companies. Therefore, future research could provide additional implications for the pharmaceutical industry if it continuously expands its importance by using worldwide data and considering other variables that can affect technological efficiency.

## Figures and Tables

**Table 1:** Sample statistics.

	Traditional Pharmaceutical Companies	Pharmaceutical Companies Diversified into the Medical Device Business
Revenue (unit: KRW)	56,448,976,777.2374	157,844,096,262.3630
	(62,455,618,526.1714)	(224,171,430,459.2680)
Total cost of sales (unit: KRW)	30,811,491,241.2451	92,076,225,212.9121
	(35,513,449,329.7149)	(146,325,067,289.8940)
Total Asset (unit: KRW)	83,909,168,301.5564	209,512,446,516.4840
	(100,265,820,064.0890)	(293,839,417,481.1320)
Total Wage (unit: KRW)	7,478,634,065.1751	19,322,657,708.7912
	(10,223,940,758.3186)	(21,378,708,878.6833)
Number of firms	103	71
Number of samples	1028	728

Note: Numbers in the parenthesis are standard deviations. 1 USD is 1,105.0 KRW (Korean Won) as of 25 January 2021.

**Table 2:** Estimation results of group and meta-frontier production functions.

	Traditional Pharmaceutical Companies	Pharmaceutical Companies Diversified into the Medical Device Business	Meta-Frontier	
	Estimate (*t*-Value)	Estimate (*t*-Value)	LP	QP
Constant	5.9381 (1.3361)	−4.9398 *** (−2.9994)	8.8697	12.3158
ln *x*_1_	1.6171 *** (6.1998)	2.1117 *** (7.9280)	1.7413	0.5321
ln *x*_2_	−0.5390 (−1.3407)	−0.3842 (−1.3342)	−0.3704	−0.7483
ln *x*_3_	−0.4458 (−1.3457)	−0.2718 (−1.0542)	−1.0931	0.3011
(ln *x*_1_)^2^	−0.0157 *** (−4.0087)	0.0836 *** (11.1918)	0.0949	0.0428
(ln *x*_2_)^2^	0.0178 (1.0392)	−0.0400 ** (−2.0595)	0.0072	0.0026
(ln *x*_3_)^2^	−0.0179 (−1.0271)	0.0440 *** (3.3018)	0.0715	0.0427
ln *x*_1_ × ln *x*_2_	−0.0357 *** (−3.0172)	−0.0281 (−1.0409)	−0.0731	0.0173
ln *x*_2_ × ln *x*_3_	0.0285 (0.9297)	0.1421 *** (5.6209)	0.0847	0.0180
ln *x*_3_ × ln *x*_1_	0.0297 * (1.9506)	−0.2101 *** (−11.0269)	−0.1683	−0.1045

Note: *, **, and *** mean *p* < 0.1, *p* < 0.05, *p* < 0.01 respectively.

**Table 3:** SFA estimates of technical efficiencies and meta-technology ratios.

		Traditional Pharmaceutical Companies	Pharmaceutical Companies Diversified into the Medical Device Business
TE	average	0.7628	0.5911
	stdev	0.1645	0.1019
	min	0.0452	0.2446
	max	0.9847	0.9848
MTR_LP	average	0.8039	0.9454
	stdev	0.1296	0.0650
	min	0.0004	0.4129
	max	1.0000	1.0000
MTR_QP	average	0.7667	0.8882
	stdev	0.1153	0.0816
	min	0.0071	0.5257
	max	1.0000	1.0000
TE*_LP	average	0.6097	0.5559
	stdev	0.1492	0.0814
	min	0.0002	0.1989
	max	0.9195	0.8365
TE*_QP	average	0.5817	0.5223
	stdev	0.1389	0.0825
	min	0.0068	0.1846
	max	0.8570	0.8513

## Data Availability

Restrictions apply to the availability of these data. Data was obtained from KIS-VALUE and are available from https://www.kisvalue.com/web/index.jsp with the permission of KIS-VALUE.
